# Programme administrators’ views on barriers in the Cuban-South African medical collaboration at UKZN

**DOI:** 10.4102/hsag.v30i0.2846

**Published:** 2025-04-09

**Authors:** Valerie Chinniah, Ashika Maharaj

**Affiliations:** 1 Discipline of Human Resource Management, College of Law and Management Studies, University of KwaZulu-Natal, Durban, South Africa

**Keywords:** human resources for health, Cuban-South African medical collaboration, medical professionals, UKZN, Department of Health

## Abstract

**Background:**

The Cuban-South African medical collaboration, initiated in the early 1990s, aims to address the shortage of medical doctors, particularly in remote and rural South Africa, thereby contributing to achieving health-related outcomes for universal health coverage.

**Aim:**

This study focusses on the perceptions of programme administrators of the systemic barriers in coordinating the Cuban-South African medical collaboration programme.

**Setting:**

The main site for this study is the University of KwaZulu-Natal, Nelson Rohihlahla Mandela School of Medicine. It is the only medical university located in KwaZulu-Natal.

**Methods:**

A qualitative exploratory design was employed to understand the barriers to coordinating the programme. Purposive sampling was used to select participants. Data collection was conducted through semi-structured interviews with participants at the medical school. Data were analysed using thematic analysis.

**Results:**

The study revealed several barriers that hinder the smooth operation of the programme, such as incomplete documentation and delays in registration, delays in equipment distribution and transportation complications because of registration issues, language barriers, connectivity issues, and the need for provisions during load shedding and water shortages.

**Conclusion:**

Addressing these barriers is crucial for improving the overall experience of the cohort students, who play a vital role in enhancing human resources for health, driving health reforms and supporting primary healthcare in South Africa.

**Contribution:**

The study provides recommendations to enhance the programme’s effectiveness. These measures are essential for ensuring the sustainability and success of the Cuban-South African medical collaboration programme.

## Introduction

The scarcity of skilled healthcare professionals is a worldwide challenge (World Health Organization [WHO] 2019). While the exact figures may vary depending on the region and the specific healthcare needs of different populations, several key factors contribute to this scarcity (Kreuter et al. 2021). Those contributing factors are population growth, an ageing healthcare workforce, geographical disparities between urban and rural areas, the migration of skilled healthcare workers from low- to high-income countries, limited training capacity in medical schools and the retention of health workforce because of poor working conditions, low salaries and high workload (Zihindulai, MacGregor & Ross 2018). Addressing this scarcity necessitates a comprehensive approach, including increased investment in healthcare education, improved working conditions, policies to retain professionals in underserved areas and global cooperation to mitigate migration challenges (Kreuter et al. 2021).

Addressing the scarcity of healthcare workers is crucial to providing access to essential medical services, which in turn improves overall health outcomes and reduces health disparities (Ayo-Yusuf 2015; Bizcommunity, 2021). Moreover, it enhances the quality of care and ensures a more effective response to population healthcare needs (Squires et al. 2020; WHO 2016). Access to healthcare services and a well-functioning healthcare system also promote social stability by tackling public health challenges and reducing inequalities, which is a fundamental human right and this contributes to the bolstering of healthcare systems both nationally and globally.

Cuba plays a significant role in addressing the scarcity of human resources for health among 67 countries worldwide through its medical cooperation programmes (Feinsilver, 2023). These initiatives involve sending Cuban medical professionals, including doctors, nurses and other healthcare workers, to countries in need, particularly in low- and middle-income regions on medical missions (Lamrani, 2021). Cuba has also been instrumental in training opportunities for students from partner countries to study medicine at Cuban universities (Feinsilver, 2023). Upon completing their studies, these students return to their home countries to contribute to their healthcare systems. Overall, Cuba’s assistance in human resources for health demonstrates its commitment to international solidarity and its contribution to addressing healthcare disparities and promoting health equity worldwide in order to reinforce global health systems and promote universal healthcare coverage (Lamrani, 2021).

In the early nineties, the South African government forged a pioneering partnership with Cuba, aiming to empower medical students hailing from underprivileged backgrounds. This visionary agreement entailed comprehensive training for these students, with the implicit understanding that upon completion of their degrees, they would return to their communities to fulfil a crucial role in healthcare service (Motala & Van Wyk, 2019). While this collaboration has tried to address the scarcity of medical professionals in South Africa it encountered various challenges in realising its ultimate goal of building up the skills and capacity of doctors in underserved areas where their presence was most critical. Motala (2014) and Mqadi (2015) have examined the Cuban-South African medical collaboration, exploring the challenges for both students and institutions. These studies focussed on comparing Cuban medical training with traditional Bachelor of Medicine and Bachelor of Surgery (MBCHB) programmes (Motala, 2014) and assessing the overall viability, feasibility and cost-effectiveness of the collaboration between Cuba and South Africa (Mqadi, 2015). However, there has been a dearth of studies that have focussed on the barriers experienced when coordinating the reintegration stage of the programme when students return from Cuba to complete their training at local medical schools (Donda, Hift & Singaram 2016; Donda, Singaram & Hift 2022; Motala & Van Wyk 2021; Phasha 2021; Squires et al. 2020).

Proper planning for human resources for health ensures the quality, effectiveness and sustainability of the health workforce, as recognised by the WHO (2016). Focussing on the barriers in managing the Cuban-South African medical collaboration is critical in ensuring the seamless operation and efficacy of the programme, particularly when coordinating the reintegration stage of the programme when students return from Cuba to complete their training at local medical schools. By addressing these barriers effectively, not only can the programme run more smoothly but also the students’ experience in becoming medical doctors can be significantly enhanced. While still in training, these medical doctors remain students at the University of KwaZulu-Natal (UKZN). They require support from the institution to continue their learning. Therefore access to Wi-Fi is critical as they still need to complete assessments and attend seminars online as they are doing training in remote locations (Decentralised Clinical Training Programme [DCTP] sites). The University of KwaZulu-Natal provides students with data for this very purpose. This is in line with the UKZN Strategic Plan 2023–2032, where an Excellent Student experience is one of its main pillars (UKZN Strategic Plan 2023–2032).

This article forms part of a main study that focussed on human resources for health policy at the macro-level, programme challenges at the meso-level and challenges facing South African students in the Cuban cohort at the micro-level. Given the significant role of the Cuban-South African medical collaboration in enhancing healthcare delivery in South Africa, it is imperative to address the administrative barriers encountered to sustain and expand the programme. Understanding and addressing administrative hurdles will optimise the collaboration, making it more effective and sustainable. Insights from the study can inform policy development in terms of Human Resources for Health and identifying and mitigating administrative inefficiencies will ensure better utilisation of resources, maximising the impact of the collaboration and ensuring that the programme coordinators can navigate regulatory and logistical complexities more efficiently. The need for a detailed study on the barriers encountered in coordinating the Cuban-South African medical collaboration will provide critical insights that can enhance this important partnership’s effectiveness, sustainability and scalability, ultimately contributing to better healthcare outcomes in South Africa and beyond.

## Research methods and design

This study employed a qualitative research approach, chosen for its suitability in understanding the barriers encountered when managing the Cuban-South African medical collaboration at the Nelson Mandela School of Medicine at UKZN. Qualitative research involves an iterative process aimed at enhancing understanding by closely examining the phenomenon under study, primarily through the collection and analysis of textual data from sources such as interviews, focus groups and participant observations (Aspers & Corte 2019). In health research, qualitative approaches are particularly valuable for comprehending human health, health services, and health behaviours and practices (Green & Thorogood 2018).

### Setting

The main site for this study is the UKZN, Nelson Rohihlahla Mandela School of Medicine. It is the only medical university located in KwaZulu-Natal and partners with the Department of Health to train returning students from the Cuban cohort and to prepare them for examination and graduation. However, the medical students’ training takes place at various DCTP sites located in various districts throughout KwaZulu- Natal. These include Ngwelazane, Lower Umfolozi, Stanger, Newcastle and Port Shepstone, and students from the Cuban cohort are required to train at one of these sites as a mandatory requirement from the Department of Health to support the principles of the primary healthcare approach.

### Study design

The exploratory study design was used to efficiently address the aim of the study and investigate the barriers encountered when managing the Cuban-South African medical collaboration at the Nelson Mandela School of Medicine at UKZN. Exploratory research is well suited for situations where deeper comprehension is sought, serving as a foundation for future investigations by providing valuable insights and aiding in the selection of appropriate design and identification of relevant variables (Aspers & Corte 2019).

### Mitigating researcher bias and enhancing reflexivity in qualitative research

A key element in safeguarding the integrity of qualitative research is the practice of reflexivity. Reflexivity involves the researcher’s ongoing self-awareness and critical reflection on how their own biases, beliefs and personal experiences may influence the research process (Olmos-Vega et al. 2022). Throughout the study, the researcher engaged in continuous reflexive monitoring, consciously acknowledging and addressing potential biases that could affect data collection, analysis or interpretation. This reflexive stance helped ensure that the research findings were as objective and trustworthy as possible, ultimately contributing to the credibility and reliability of the study. Unconscious bias on the part of the researcher poses a significant risk to the accuracy and validity of qualitative research findings (Green & Thorogood 2018). Such biases, often socially embedded and culturally ingrained, can lead to skewed interpretations that do not accurately reflect the participants’ true experiences or perspectives (Olmos-Vega et al. 2022).

Adopting a detached, emotion-free perspective was essential to maintaining objectivity. The researcher consciously worked to approach the subject matter without preconceived notions thereby reducing the likelihood of unintentionally influencing participants’ responses. In order to get an authentic and uninfluenced response, questions were carefully crafted and delivered in a neutral tone, allowing participants the space and time to express their thoughts and experiences in their own words. The use of digital communication platforms, such as Zoom and WhatsApp, for conducting interviews proved to be particularly advantageous. These platforms offered a comfortable and familiar environment for participants, which likely contributed to a sense of ease and openness during the interviews. By interacting with participants in a setting where they felt safe, the researcher was able to gain a more genuine dialogue, which contributed to obtaining rich data.

### Participants

The sample included five programme administrators involved with the administration of the Cuban cohort when they returned to the Nelson R Mandela School of Medicine at the UKZN and studied in Cuba between 2018 and 2022. Because these participants were part of the wider study involving interviews with programme managers and South African students, the purpose of including the programme administrators was to provide a balanced perspective on the administrative processes and support systems.

### Sampling

Purposive sampling is a non-probability sampling technique where the researcher selects participants based on specific characteristics or criteria that are crucial to the research questions and objectives. This method allows for a deliberate and strategic selection of participants who can provide rich, detailed and relevant data, thereby enhancing the depth and quality of the research findings (Jackson & Bazeley, 2019). In this study, purposive sampling was employed to target programme coordinators at the Nelson R. Mandela School of Clinical Medicine at UKZN. These coordinators were chosen because of their direct involvement and expertise in managing the programme, making them uniquely qualified to offer valuable insights pertinent to the research objectives. The selection criteria focussed on those coordinators with first-hand experience and substantial knowledge about the programme, ensuring that their perspectives would significantly contribute to addressing the study’s aims. Given the structure of the programme, only five coordinators are directly responsible for its management. This small, specific group was identified as having the most relevant experience. Consequently, all staff members actively engaged with the Cuban cohort groups were invited to participate.

### Data collection

**FIGURE 1:**
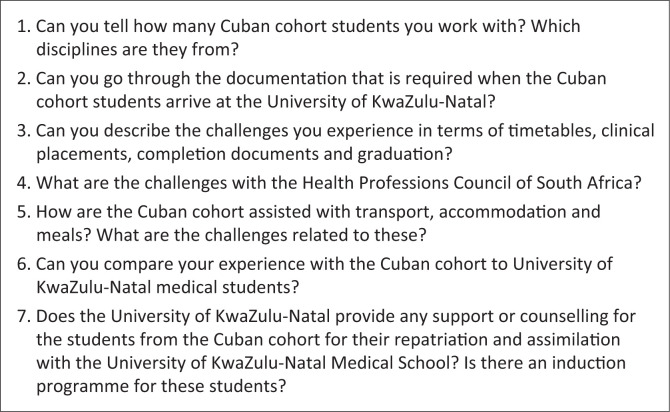
Interview schedule for programme coordinators.

Interviews were conducted using a standardised semi-structured interview protocol, which was piloted and refined prior to the study. The interview schedule was reviewed by a member of staff who served as a committee member dealing with the Cuban-South African medical collaboration programme at UKZN. This person has been involved in meetings with the Department of Health to repatriate the South African students from the Cuban cohorts to local medical schools. While this staff member agreed to evaluate the research instrument, they did however decline the request to be interviewed as part of the study because of a conflict of interest. Their recommendations included rearranging the sequence of the questions for the programme coordinators to capture the chronological sequence of events. No additional changes are required, for instance in terms of the language and wording of the questions. This protocol included open-ended questions designed to elicit detailed responses about the administrative processes. To ensure privacy and confidentiality, all interviews were conducted in private settings, via secure online Zoom platforms. Interviews were conducted with participants while they were at home or in their offices, in other words, wherever they felt most comfortable sharing their experiences. Participants were assured that their responses would be anonymised and that no personally identifiable information would be disclosed. Responses from participants were uploaded to NVivo to organise the data into themes for analysis. Interviews were conducted over a 4-week period from March 2023 to April 2023, because of the extremely tight schedules of the participants. The interviews took on average between 30 min and 60 min to conduct (Figure 1).

### Data analysis

Thematic analysis was used to analyse the interview data. This involved coding the data, identifying key themes and synthesising the findings to draw meaningful conclusions about the students’ experiences and the challenges they faced. All interview transcripts and research notes were collected and stored securely. Interviews were audio-recorded with participants’ consent to capture their responses accurately (Jackson & Bazeley, 2019). Audio recordings of interviews were transcribed verbatim to create written records of the interviews. Transcriptions were checked for accuracy and completeness to ensure reliability. All digital data, including transcripts and documents, were stored in password-protected folders on secure servers. Confidentiality of participants’ information was maintained throughout the study. Personal identifiers were removed or anonymised from transcripts to protect participants’ privacy. In this study, the data were transcribed and coded manually by reading and re-reading the transcripts highlighting the main points, comparing responses and concentrating on the insights of the participants. Originally, thematic analysis using manual coding was considered to be the most suitable method of data analysis for the study. After the initial analysis, it was decided to use NVivo to enhance the thematic analysis to add depth to the findings of the study. According to Jackson and Bazeley (2019), the use of NVivo as a means of analysing qualitative data is related to the research purposes of the study. NVivo was used to organise the data collected during the interviews, information was entered according to the themes derived from manual coding. This programme organised the data and relevant interview quotes into themes and sub-themes that emerged from the analysis. NVivo offered automated functionality for deeper analysis, including word frequencies, commonalities and comparisons. (Chinniah, 2024).

### Trustworthiness

Qualitative research uses the following as measures of trustworthiness: credibility, transferability, dependability and confirmability. These measures establish the integrity of the research and establish the value and authenticity of the data collected. The credibility of this study was established using participant review and cross-checking of the data by the researcher helped to reduce individual biases while the accuracy of the data was maintained through the use of field notes. In addition, triangulation, independent coding and verifying the true significance of the results from matching field notes enhanced the credibility of the study. The transferability in this study was established by comparing sufficient data with the data description, ensuring the applicability and generalisation of the findings. The dependability of the findings was achieved by auditing the data, preserving raw material and allowing the participants to review the data. There was also a process of manual coding and then the use of NVivo to assign codes according to the data. The confirmability in this study was achieved through maintaining meticulous records throughout the study and keeping organised details and field notes in a research journal allowed the researcher to check and recheck data, even in the analysis.

### Ethical considerations

Ethical clearance to conduct this study was obtained from the Humanities and Social Sciences Research Ethics Committee of the University of KwaZulu-Natal (reference no.: HSSREC/000002464-2021) prior to data collection. Informed consent was obtained from all participants, and confidentiality and anonymity were ensured throughout the study.

## Results

### Sociodemographic characteristics

A total of five programme coordinators were interviewed: four females and one male. The participants included three Indians, one white and one black individual. All the participants had held their positions for more than 10 years (Table 1).

**TABLE 1:** Demographic information of participants.

No.	Alias	Designation	Gender	Race	Tenure (year)
1	PA_CP	Administrator	Female	Indian	> 10
2	PA_DP	Administrator	Male	Indian	> 10
3	PA_MM	Administrator	Female	Indian	> 10
4	PA_SS	Administrator	Female	White	> 10
5	PA_SM	Administrator	Female	Black	> 10

The data analysis revealed four key themes. In Table 2, these themes are presented along with a brief synopsis and accompanying data from the transcripts. The frequency with which these themes were stated in the five interviews implied their common occurrence and emphasised their significance in the study.

**TABLE 2:** Themes and sub-themes.

Theme	Sub-themes
1. Organisational-level barriers affecting reintegration of Cuban cohort students	-
2. Logistical and transportation constraints	2.1. Delays in the distribution of medical equipment 2.2. Increased costs of transportation
3. Psychological and social barriers experienced by Cuban cohort students	3.1. Language barriers experienced by Cuban cohort students 3.2. Personal dilemmas faced by Cuban cohort students
4. Difficulties experienced by Cuban cohort students at DCTP sites	-

DCTP, Decentralised Clinical Training Programme.

### Theme 1: Organisational-level barriers affecting the reintegration of Cuban cohort students

This theme looks into the organisational-level barriers that affect the reintegration of Cuban cohort students into the university. The challenge stems from students’ failure to complete the mandatory documentation while in Cuba, leading programme coordinators to wait for the students’ return to South Africa to finalise their registration at the UKZN.

A participant stated that, preferably, students should be registered in the system before reaching South Africa, recognising the challenges this may involve:

‘That was causing them a major hassle and undergrad office assisted there because the students that couldn’t get it [*paperwork*]done in Cuba and had to come to South Africa to do it at the undergrad office and they helped cos some of them didn’t have access to the electronic systems that we have.’ (PA_MM, 42 years old, Female)

This hindrance caused considerable disruption, motivating the undergraduate office to help students who could not complete their documentation in Cuba because of limited access to electronic systems. The lack of necessary technological infrastructure, such as computers, scanners or printers, in Cuba, created a major challenge, stopping students from completing their registration. Both manual and electronic forms are crucial for updating the university database with student information. However, the interviewees noted that students from Cuba found this process especially problematic. Their effort in completing the documentation correctly without assistance from the undergraduate office led to delays, compelling some students to return to South Africa to process their documentation. The challenges faced were highlighted by a participant:

‘The forms were not completed correctly, you know. So, those forms could not be used. So as a result now we wait for them to get here and then we give them, but the fact that you have to wait for them to get here and do the forms, it causes a delay in their registration.’ (PA_SM, 34 years old, Female)

One can therefore deduce that the organisational barriers related to incomplete documentation emphasise the complexity of registration and data alignment, highlighting the need for proactive measures to simplify the documentation process, especially for students in remote areas like Cuba. As a result, the delayed registration of Cuban cohort students as UKZN students posed challenges for coordinators in processing stipends on time.

This delay had a profound impact on the students’ ability to meet basic needs, a participant, elucidated:

‘Once registration is delayed, it delays the whole, a lot of other things like, the release of their stipends. You know from the moment they get here they are hungry, they need money, you know, but those refunds can’t be processed, I mean, before they are active on the system. So those are the challenges that pertain to the completion of documents.’ (PA_SM, 34 years old, Female)

Furthermore, delays in processing exam results by the Department of Health, coordinated through the Cuban Council’s central offices at both provincial and national levels, contributed to delays in registering students at the UKZN. In line with the Health Professions Council of South Africa’s (HPCSA) regulations, final-year students must complete specific documentation to register with the Department of Health as junior interns. It includes capturing and paying for the registration, which allows students to begin clinical rotations, as well as capturing the completion certificate needed for registration with the Department of Health and getting a licence to practise medicine from the HPCSA:

‘The final exam results are often delayed because of the state exam. We at UKZN do not have any input in that exam. We don’t even host that exam, so we can’t expedite the release of results because we don’t have access to those results.’ (PA_MM, 42 years old, Female)

Adding to the complexity, the HPCSA’s offices are located in Pretoria. Therefore, physical copies of documents from the UKZN must be couriered in batches to the Council’s offices. A significant issue is the presence of a staff member responsible for processing applications from the UKZN, often resulting in documents being lost or misplaced. PA_DP highlighted this issue:

‘What happens is that Health Professions Council of South Africa is based in Pretoria so once we receive the student information, completed forms with the supporting documents, it has to be couriered to Health Professions Council of South Africa. We send them off in bulk and depending on who’s … Health Professions Council of South Africa doesn’t have designated staff for these. So whoever is on call that day, will collect the forms, then sometimes these forms are left in out trays, in trays and only after follow up, when we follow up and follow up then these forms are located. There are a whole lot of delays in administering these forms.’ (PA_DP, 45 years old, Male)

In summary, delays in stipend processing and interactions with regulatory bodies highlight the complex challenges coordinators face in ensuring the seamless operation of the programme for Cuban cohort students.

### Theme 2: Logistical and transportation constraints

This theme addresses the logistical and transportation challenges encountered by the Cuban cohort upon their return to South Africa. Two key sub-themes explore the difficulties UKZN coordinators face in obtaining and delivering critical goods and services for the cohort.

#### Sub-theme 2.1: Delays in the distribution of medical equipment

The orientation programme for Cuban cohort students covers essential information on accommodation, transport and the distribution of personal protective equipment (PPE). However, operational challenges arise in these areas. Distributing equipment and protective gear – including lab coats, stethoscopes, patella hammers and laptops provided by the Department of Health – is hampered by delays in the registration system. Poor organisation and ineffective planning by the Department of Health, combined with ineptitudes within the Cuban Council’s central coordinator’s office, create additional workloads and resource wastage for UKZN coordinators. Subsequently, coordinators must arrange the pickup or delivery of PPE to clinical sites, as students cannot begin their practical training without these critical items.

A participant reflected on this issue:

‘The registration delays cause students to arrive at different times and this makes it hard to give them their PPEs as well as the gear they will need at their facilities. Even getting the DOH laptops to them becomes an issue as often these arrive after the students have left to the DCTP sites.’ (PA_MM, 42 years old, Female)

‘Then getting them back to collect and start is an issue because some of them don’t come back on time, then they not based in Durban directly. Depending which rotation they doing, they could end up in Ngwelezane, Newcastle, Stanger, Port Shepstone or if you are doing public health you could end up in one of the rural sites for your rotation so they not like coming back to Durban to Med School to collect these stuff. You must remember that all these things are procured by DOH they have to be collected by the students in person, to sign and ensure that it is received and things like that.’ (PA_CP, 32 years old Female)

#### Sub-theme 2.2: Increased costs of transportation

The transportation of medical students to remote clinical sites presents a major challenge for UKZN coordinators, particularly when managing large groups travelling to the 10 DCTP sites. These sites are distributed across areas such as Stanger, Port Shepstone, Empangeni, Queen Nandi, Newcastle, Pietermaritzburg and public hospital facilities in Durban:

‘Ja there is quite a few problems like missed transport. Also the outlying areas students have to carry all their luggage, all their necessities, all their bedding and whatever else they taking to that res cos they won’t just be there for six weeks, they’ll be there for a semester which is 6 months of the year so if you miss all … basically we arrange buses for the students and trucks for their luggage so if they miss their transport it’s a bit of a problem because the cost for one student and let’s say fifteen students is the same. Our costing is per student. Whether we use the bus for one student or sixty students it’s the same price so our service suppliers are on that mandate that they can charge per trip so it becomes a problem based on that and we have to get permission from faculty office, from Prof B’s office to ensure that everything to arrange this.’ (PA_DP, 45 years old, Male)

The unavailability of designated vehicles and staff at certain sites, particularly in remote areas with limited public transportation, often requires dependence on local taxi operators. However, because these operators typically operate on a cash-only basis, this creates challenges for the university’s financial procedures:

‘In the smaller rural sites it is very difficult because you find that we need to arrange with the local taxi people and the local taxi people they operate on cash basis so, you know the university cannot pay cash rather you render the service and then you invoice us. Even if they do want to assist us you find that the university requires vendors to have some documents that are required like a tax clearance certificate, banking accounts, and all of that. So, you find that those local taxi people, don’t have those documents as a result even if they want to assist us then they won’t be able to invoice us because of the documentation.’ (PA_SM, 34 years old, Female)

Despite these challenges, the UKZN demonstrates effectiveness in supporting students, particularly by arranging transportation for family emergencies and other urgent situations. This initiative is designed to offer extra support and assistance to medical students who may be away from their families during their training:

‘I mean UKZN transport to clinical facilities. We provide them with transport and err we assist with transport in cases of emergency like maybe if students is, is, is, is sick or maybe family crisis. You know sometimes they will ask err maybe you need to come err err, go home you know for family emergencies. We would assist with that where we can, where we can and in some instances we assist them, we take them err for shopping but this is only applicable in the sites where we have UKZN vehicles and staff allocated cos not all the sites are allocated with UKZN staff and vehicles. For, for, for for the smaller sites like the rural sites, where we place a fewer number of students you will find that we don’t have any err … UKZN person there or UKZN vehicle.’ (PA_DP, 45 years old, Male)

### Theme 3: Psychological and social barriers experienced by Cuban cohort students

This theme focusses on the multifaceted psychological and social barriers faced by Cuban cohort students upon their return to South Africa, as revealed in the thoughtful responses of programme coordinators. The key social challenges include language barriers, communication difficulties and the psychological effects of the difficulties encountered by the students.

#### Sub-theme 3.1: Language barriers experienced by Cuban cohort students

Language arose as a key feature of the social challenges encountered by coordinators at clinical sites, influencing both university offices and hospital environments. These linguistic barriers have an effect on not only the students’ communication with coordinators but also their communication with patients. Another participant highlighted this concern:

‘It was also their language that they couldn’t understand. For example, if there was a Zulu-speaking patient at the hospital and they had to go clerk that patient, the students had problems understanding what the patient was saying so there always had to be an English-speaking student or consultant or a doctor there with them in order for them to understand the teaching.’ (PA_CP, 32 years old, Female)

Although Cuban cohort students have a solid foundation in medical knowledge, challenges arise when translating this knowledge into English. This difficulty stems from their medical education being conducted in Spanish, combined with the fact that English is not their native language.

#### Sub-theme 3.2: Personal dilemmas faced by Cuban cohort students

Assimilation to the UKZN culture presents a psycho-social challenge for Cuban cohort students, testing coordinators’ ability to help them navigate university processes. Coordinators regularly help students not only with academic concerns but also with personal issues, despite missing formal training in this area. Encouraging students to seek assistance from Student Support Services or Academic Development Officers is critical. However, many students are unwilling to share personal challenges, obfuscating the support process.

A participant emphasised the prevalence of mental health issues among students, further aggravated by their reluctance to engage with counsellors:

‘Students are often embarrassed or shy to share their problems with counsellors. They fear stigmatisation or isolation, or just being treated differently to their peers … yeah it makes our jobs much harder.’ (PA_SM, 34 years old, Female)

Additionally, the arduous academic workload and demanding schedules often limit students’ availability to access support services during regular hours, requiring coordinators to provide assistance after hours. Another participant elaborated on this challenge:

‘So, the programme itself was quite long. We would start at eight o’clock and finish at four, four thirty. Quite a long day, digitally it’s impossible to stay focused and alert. Err, the students maybe had an hour break. We saw them continue throughout their studies, throughout their blocks. They would have choc-o-block blocks in the sense whether it’s full online or blended learning out into the ward rounds and coming back to tutorials in the afternoon. They were constantly busy and that prevented us from having direct access to them during their blocks. So if a student needed to access student services, if would have to be in the evening.’ (PA_SS, 35 years old, Female)

The absence of private spaces for students to have confidential conversations with counsellors adds another layer of complexity, limiting the efficiency of psycho-social support. A compelling illustration of this challenge was offered:

‘If we do get permission to consult with them during the day, they don’t have a confidential space to consult. They would either be in the work, ward rounds, they, I had a student sitting in the bathroom, whispering to me on their cellphone and WhatsApp video call and I looked at them and you could see the tiles behind them and you would say, you know, are you in the bathroom?’ (PA_SS, 35 years old, Female)

In 2019, the coronavirus disease 2019 (COVID-19) pandemic added further challenges for programme coordinators managing the Cuban cohort. Concerns around the stigma associated with a positive COVID-19 diagnosis, motivated some students to withhold their test results, presenting possible risks to both peers and staff. This situation had a wider impact, increasing anxiety among fellow students, some of whom, worried about their well-being, discreetly reported such cases to programme coordinators.

It was revealed:

‘It would now cause panic to other students because they would see that, you know, the student has got COVID signs but they are kind of like still attending classes and whatever and whatever. And the student would report the matter but now you can’t, because they would say, please I’m reporting this in confidence so I don’t want my name to be mentioned.’ (PA_SM, 34 years old, Female)

Moreover, COVID-19 restrictions prevented coordinators from conducting workshops designed to help students acclimate to the programme, disrupting an important aspect of their support strategy.

It was explained:

‘It was very tough with COVID-19. These kids were left to their own devices basically. We were unable to hold in person workshops to help acclimatise them to their new environments. At that stage we were still figuring out the logistics of online workshops etc.’ (PA_SM, 34 years old, Female)

### Theme 4: Difficulties experienced by Cuban cohort students at Decentralised Clinical Training Programme sites

This theme highlights the various challenges related to load shedding, Internet connectivity and infrastructure issues, such as water shortages. To address these issues, ongoing projects were launched to install water tanks and solar power systems, representing a substantial investment. PA_SM detailed the mitigation measures implemented, including equipping JoJo tanks with pumps and supplying rechargeable solar energy panels at rural sites.

A participant explained that:

‘The challenges with poorly developed infrastructure such as piped water went beyond what we [*coordinators*] could control. Students would have to use JoJo tanks or even bore holes for water … basic hygiene became a problem especially in the far out rural areas. We got funding to start projects to install infrastructure for water collection and solar power.’ (PA_CP, 32 years old, Female)

These constraints significantly disrupted the registration process, academic access and overall experience of the Cuban cohort students returning to the UKZN. Limited Wi-Fi connectivity and insufficient access to data emerged as major obstacles, adding pressure on students already striving to catch up. To mitigate these challenges, coordinators implemented temporary measures, such as providing routers, until more permanent solutions could be established. Reliable connectivity was essential for key tasks like registration and accessing the university’s databases.

Another participant stated that:

‘The Internet connectivity was such a problem. Remote locations meant poor connectivity as the service provider network towers were often few and far between.’ (PA_SS, 35 years old, Female)

Moreover, the challenges extended beyond academics, hindering students’ ability to reach Student Support Services and Academic Development Officers, which further limited access to psychosocial support. Unstable Wi-Fi networks, particularly in rural areas, compelled coordinators to develop contingency plans, as noted by a participant:

‘We found that Zoom after hours, on the residence Wi-Fi is terrible because everyone is on Wi-Fi at that time. So, if there is a break in the, the Wi-Fi connectivity, we have our plan B which is a WhatsApp video call, which is a lot easier on the system.’ (PA_SS, 35 years old, Female)

In the ‘Discussion’ section, a synthesis between the findings of this study and the current literature occurs.

## Discussion

The principal findings of the study reveal significant barriers to the smooth running of the programme. Incomplete and incorrect forms led to delays in registration, impacting students’ ability to register with the HPCSA and causing delays in receiving stipends and clinical site allocations. This also postponed the distribution of equipment and PPE. Logistical issues and additional transportation costs arose because of these delays. Students faced difficulties repatriating and assimilating with UKZN MBChB students and local patients, compounded by language barriers. Coronavirus disease 2019 concerns led to self-isolation without disclosure, resulting in missed classes and unmet needs for personal and psychological support. Coordinators struggled with Wi-Fi access, Internet connectivity and provisions during load shedding and water shortages at remote sites. These challenges significantly impacted the students’ education and well-being.

Donda et al. (2016) as well as Mabunda et al. (2023) highlight that the primary challenge faced by the Cuban cohort is the process of repatriation to South Africa. The challenges within the Cuban-South African medical collaboration are multifaceted, encompassing the processing of students’ documentation, administration involving the Department of Health and HPCSA, operations in terms of transport, facilities and accommodation, and social and technical issues. This study’s findings indicate that these issues pose additional challenges and delays in the successful completion of medical training for students in the Cuban cohort. Understanding the challenges faced by programme coordinators offers valuable data to address these obstacles and enhance the overall experience within the Cuban-South African medical collaboration.

### Organisational-level barriers affecting the reintegration of Cuban cohort students

This theme reveals the complex processes and challenges encountered by coordinators at the Nelson Mandela School of Medicine, UKZN in managing South African students within Cuban cohorts. It focusses on two key aspects: processing student documentation and overseeing programme administration. These elements provide insight into the initial phase of the administrative process as Cuban cohort students’ return to South Africa to complete their medical training at the University.

The delays in student registration at UKZN trigger a cascade of administrative issues. For instance, the processing of stipends and other essential administrative tasks is contingent upon active registration status. This delay impacts students’ ability to meet their basic needs and hampers their academic progress. Beyond registration, the administration of the programme involves coordinating various aspects of student support and academic integration. These findings are supported by Phasha (2021) who found that coordinators at South African medical schools encounter complex procedures in processing documentation and managing the integration of returning students. These administrative hurdles can lead to delays and confusion during the reintegration process. Programme coordinators face significant issues in aligning the academic schedules and curricula of the Cuban medical training with those at UKZN. This misalignment can result in gaps in knowledge and skills, requiring additional support and bridging courses to ensure students meet the South African medical education standards. These findings are supported by Donda et al. (2016) who found that:

[*T*]he Cuban medical curriculum emphasizes health promotion and disease prevention, whereas the South African system focuses more on a hospital-based biomedical model. This disparity necessitates additional training and adaptation for returning students to meet local clinical requirements. (p. 2)

Moreover, backlog issues in processing exam results and regulatory documentation by the Department of Health and the HPCSA exacerbate administrative delays, further impeding students’ registration and progression within the programme. These delays not only affect students’ academic timelines but also their eligibility for professional certification and placement in medical internships and residencies. According to Bizcommunity.com (2023), the HPCSA has acknowledged these delays and is working to address them.

These issues at UKZN highlight the need for several strategic interventions in terms of enhanced technological infrastructure, improved administrative support, harmonised academic frameworks to align curricula and streamlining the processes for exam result verification and regulatory documentation through better coordination with the Department of Health and HPCSA. This should reduce the backlog and facilitate timely student progression.

### Logistical and transportation constraints

Despite outlined plans for equipment distribution during orientation programmes, operational hurdles arise because of delays in student registration and poor coordination between the Department of Health and the Cuban Council’s central coordinator’s office. These findings are supported by Donda et al. (2016) and Mabunda et al. (2023) who identified these as major challenges in their studies. University coordinators must navigate these challenges to ensure the timely provision of necessary equipment and resources for students, often resorting to alternative arrangements to address logistical constraints.

The distribution of essential equipment is a critical aspect of ensuring that students are well-prepared for their medical training. However, delays in the registration system can cause significant bottlenecks. Without active registration, students may not receive their equipment on time, impacting their ability to participate fully in their training. Furthermore, the poor coordination between the Department of Health and the Cuban Council’s central coordinator’s office exacerbates these delays. University coordinators often have to find alternative solutions, such as borrowing equipment from other departments or sourcing from external suppliers to ensure that students are not left without the necessary instruments. These bottlenecks were also identified as major issues by Bizcommunity (2023).

Transportation poses another significant operational challenge for programme coordinators, particularly in coordinating transport to outlying rural clinical sites. Limited access to designated vehicles and staff at remote sites necessitates reliance on local taxi operators, further complicating transportation arrangements. This reliance introduces variability and unpredictability in transportation, making it difficult to ensure consistent and timely travel for students. Financial constraints and operational inefficiencies in the use of cash-based systems for transportation payments present additional hurdles. Donda et al. (2016) and Squires et al. (2020) found similar issues in their studies. Coordinators must align with the financial policies of the University while managing cash transactions, which can be prone to delays and inaccuracies. The constraints of ensuring compliance with financial policies while managing the practicalities of cash payments add another layer of complexity to the operational management of the programme.

Despite these challenges, coordinators demonstrate resilience in ensuring students’ access to clinical sites and addressing emergency transport needs. Their commitment to supporting students’ academic and professional development is evident in their proactive approach to problem-solving and their willingness to go beyond their standard duties to facilitate smooth operations.

### Psychological and social barriers faced by Cuban cohort students

Language barriers are a primary social challenge that affects both academic and clinical settings. Effective communication between students and coordinators, as well as between students and patients, is crucial for the success of the programme. Several studies (Donda et al. 2016; Motala & Van Wyk, 2016; Phasha 2021; Torres et al. 2021) highlight language barriers as a significant issue in Cuban medical training. This study confirms that even at the programme level, language issues hinder effective communication between coordinators and students.

The COVID-19 pandemic exacerbated these communication barriers. The need for remote communication and the increased reliance on digital platforms, where language barriers are more pronounced, intensified the difficulties faced by students and coordinators. This situation highlighted the necessity for robust communication support mechanisms to mitigate the impact of language barriers (Torres et al. 2021).

The psychosocial well-being of students is another critical area of concern. The pandemic amplified mental health issues among students, highlighting the inadequacies in the existing support systems. Students often feel reluctant to disclose personal issues because of a lack of confidence in the confidentiality and effectiveness of the support services available. Limited access to confidential spaces for counselling further restricts students’ ability to seek help.

The breakdown in communication regarding personal issues is particularly concerning. Dramowski et al. (2020) emphasise the need for a ‘workforce preservation’ approach in South Africa to prioritise the health and safety of healthcare workers, including medical students. Adopting this approach during training can create a safer environment for students to disclose their concerns and fears to coordinators.

Assimilation into UKZN’s culture presents additional psycho-social challenges for Cuban cohort students. These challenges are compounded by academic pressures and workload demands. These findings are supported by the 2022 study by Donda, Hift and Singaram. Programme coordinators often go beyond their regular duties to provide support and assistance to students, but systemic deficiencies in support services hinder their efforts. Limited access to support services and confidential spaces for counselling sessions pose significant barriers for students seeking psycho-social support.

The need for enhancing student support and well-being is underscored by Donda et al. (2016, 2022) as well as Torres et al. (2021), who emphasise the importance of robust support systems in promoting student success and well-being. Addressing these psycho-social challenges requires a comprehensive approach that includes improving access to counselling services, providing confidential spaces, and fostering an environment of trust and openness. Social challenges are twofold dealing with communication issues and psycho-social support.

### Difficulties experienced by Cuban cohort students at Decentralised Clinical Training Programme sites

Load shedding and unreliable Internet connectivity are major obstacles for South African students returning from Cuba, particularly those in rural areas. These technical disruptions can severely affect their ability to access online learning materials, participate in virtual meetings and maintain consistent communication with programme coordinators. According to Lamrani (2021), while Cuba has become a global benchmark for thriving amid challenges and limited resources, South African students often find the transition back home challenging because of pervasive technical issues.

Programme coordinators often need to devise temporary solutions to mitigate the effects of these technical disruptions. For instance, supplying students with routers and implementing backup communication methods such as mobile hotspots and offline resources are common practices. These measures, while effective in the short term, are not sustainable and do not fully address the underlying issues. Ongoing projects aimed at improving infrastructure are critical to addressing these long-term issues. Initiatives such as installing water tanks and solar power systems are steps towards enhancing the resilience of the programme. These infrastructure improvements are designed to provide more reliable access to essential services, thus reducing the impact of load shedding and connectivity issues on students’ academic performance and overall programme experience. These findings were similar to those of Donda et al. (2022).

Despite these efforts, the inconsistent availability of electricity and Internet services hampers students’ ability to engage fully with their studies and professional development activities. These technical issues can lead to delays in completing assignments, difficulties in accessing research materials, and interruptions in communication with peers and mentors. Ross et al. (2024) support these findings and find that addressing these problems requires a multi-faceted approach that includes both immediate and long-term strategies. Significant investment in infrastructure, particularly in rural areas, is essential (Chinniah, 2024). This includes expanding access to reliable electricity and Internet services and enhancing the robustness of these systems to withstand disruptions. Implementing renewable energy solutions such as solar power can provide a more reliable and sustainable source of electricity. According to De Villiers et al. (2017) and Ross et al. (2024), these types of solutions can help mitigate the effects of load shedding and ensure continuous access to power for academic and professional activities. Partnering with Internet service providers to improve connectivity in rural areas can enhance students’ ability to access online resources and communicate effectively with coordinators and peers. Providing subsidised Internet packages for students may also be a viable short-term solution (Chinniah, 2024).

By addressing these issues through targeted interventions, the Cuban-South African medical collaboration programme can enhance its resilience and ensure that students have the support and resources they need to succeed academically and professionally. This holistic approach will contribute to the sustainability and effectiveness of the programme, benefiting both students and the broader healthcare system. These conclusions are supported by recent studies (Donda et al. 2022; Ross et al. 2024). This study found that challenges faced by South African-Cuban cohort students across the different training sites were similar to those described in other studies. Therefore, the recommendations of the study are particularly important as they focus on higher-level interventions which are beyond the programme level.

## Recommendations

The aim of this study focussed on the systemic barriers facing the Cuban-South African medical collaboration, providing a comprehensive analysis to help decision-makers and policymakers enhance the programme’s effectiveness and sustainability. By identifying and addressing these barriers, the study seeks to offer practical mechanisms and strategic recommendations to improve collaboration, thereby ensuring better health outcomes in South Africa and amplifying international medical partnerships.

Overall language training and communication improvements are essential in making the collaboration more effective. Cultural and linguistic integration programmes for South African students in the Cuban cohort are recommended as an ongoing support mechanism to orientate and help students assimilate from start to finish.

In an effort to consolidate diplomatic ties between South Africa and Cuban Health and Education ministries to ensure ongoing alignment and address issues promptly. This will help improve communication between the stakeholders responsible for the effective and efficient functioning of the programme. Both ministries need to work together to align policies and create mutual recognition agreements. This should assist with compliance with regulatory standards, reducing delays and barriers for students and doctors, and ensuring that professional qualifications are recognised. The South African Department of Health and the Cuban Department of Health need to work together to harmonise regulatory requirements, including the granting of Medical licences, accreditation standards and certification processes.

By implementing these mechanisms and recommendations, decision-makers and policymakers can address the systemic barriers of the Cuban-South African medical collaboration, ensuring its sustainability and maximising its positive impact on healthcare in South Africa. The programme is critical in enhancing human resources for health by increasing the number of medical doctors in South Africa who can serve underprivileged communities; therefore, it is definitely worthwhile.

### Limitations of the study

The findings may be subject to subjective interpretation of the researcher. This is inherent in qualitative studies. In order to mitigate the biases, the overall study included a broader participant pool to include a range of programme managers and students to provide a more comprehensive understanding of the systemic barriers of the Cuban-South African medical collaboration. However, this article was only based on one aspect of the overall stakeholders involved in the study.

Additionally, the study’s scope is limited to the perspectives of coordinators at a single institution, which may not fully capture the diversity of experiences within the Cuban-South African medical collaboration at a national level. The study design, being qualitative and exploratory in nature, may introduce certain biases. Firstly, the selection of participants through purposive sampling may have led to bias if certain perspectives or experiences are overrepresented while others are under-represented. Secondly, participants may have provided socially desirable responses or withheld sensitive information, particularly when discussing issues related to documentation or programme administration, especially in light of the current restructuring processes happening at the university. Financial and time constraints did not allow for travel to Cuba to interview students. However, these students do return to South Africa once their studies are over. The purpose of the article was to examine the integration and reintegration processes of students in the programme from a programme coordinator’s perspective.

Maintaining reflexivity throughout the research process and employing rigorous methodological techniques, such as member checking or peer debriefing, helped to enhance the credibility and trustworthiness of the study findings by validating interpretations and ensuring data accuracy. Finally, transparency in reporting, including detailed descriptions of the study design, data collection methods and analytical procedures can facilitate the identification and mitigation of biases by enabling readers to assess the validity and reliability of the research findings.

## Conclusion

The primary objective of this study was to identify systemic barriers in managing the Cuban-South African medical collaboration at the Nelson R. Mandela School of Clinical Medicine, UKZN. The research discovered critical concerns requiring immediate intervention to augment efficiency at both managerial and administrative stages.

Timely resolution is essential to rationalise admission and registration processes, enabling smoother progression throughout the training period. Proactive measures in this regard are expected to yield positive outcomes, mitigating social challenges identified in the study. Notably, societal factors, including challenges posed by the COVID-19 pandemic, have significantly impacted the programme. Addressing these gaps can substantially improve student performance and overall satisfaction.

Recognising the pivotal role of the Department of Health in driving health reforms in South Africa, it is crucial to engage them more extensively, particularly concerning preventative medicine and primary healthcare. Such collaborative efforts will not only address the barriers highlighted in the study but also permit the returning cohort to play a more active and informed role in shaping South Africa’s healthcare landscape.
